# Role of biochar in biodegradation of nonylphenol in sediment: Increasing microbial activity versus decreasing bioavailability

**DOI:** 10.1038/s41598-017-04787-2

**Published:** 2017-07-05

**Authors:** Guanghuan Cheng, Mingyang Sun, Jingrang Lu, Xinlei Ge, Huihui Zhang, Xinhua Xu, Liping Lou, Qi Lin

**Affiliations:** 1 Jiangsu Key Laboratory of Atmospheric Environment Monitoring and Pollution Control (AEMPC), School of Environmental Science and Engineering, Nanjing University of Information Science &Technology, Nanjing, 210044 People’s Republic of China; 2 Department of Environmental Engineering, Zhejiang University, Hangzhou, 310029 People’s Republic of China; 3 Office of Research and Development, U.S. Environmental Protection Agency, Cincinnati, OH 45220 USA

## Abstract

The observed strong sorption of hydrophobic organic contaminants (HOCs) to biochar presents potential implications for HOCs bioavailability and bioaccessibility in sediments, while biochar could impact sediment microbial ecology. However, the comprehensive study on the effects of biochar on HOC biodegradation coupled with bioavailability and microbial ecology are rarely documented. In this paper, the effects of biochar on the biodegradation of nonylphenol (NP) were investigated using 3 different NP concentrations (20, 50 and 500 mg/Kg) in sediments amended with different percentage of rice straw biochar (RC). Results showed that the influence of RC on NP biodegradation varied with different NP concentrations. At low NP concentrations, RC suppressed NP biodegradation by reducing NP bioavailability, while at high NP concentrations, moderate RC addition promoted biodegradation by reducing toxicity of NP to microbes. The effects of NP on microbial community structures were significant (*P* < 0.01), but those of RC were not significant (*P* > 0.05). The RC affected microorganisms through altering NP toxicity, microbial quantity and activity, but not microbial community structures. This study indicated that there could be an optimal biochar percentage in biochar-sediment systems at different HOC concentrations, which strengthened HOC biodegradation process and accelerated biodegradation rate, forming adsorption-biodegradation coupled bioremediation.

## Introduction

The ability of biochar to sorb in a nonlinear fashion to hydrophobic organic contaminants (HOCs) is well established^1, 2^. Much of current research has emphasized the important role of carbonaceous materials in reducing contaminant aqueous availability and, consequently, biodegradation and/or bioaccumulation of sediment-bound contaminants, for the purpose of remediation^3–5^. Sopeña *et al*.^6^ demonstrated how HOC desorption processes in the presence of biochar are intimately related to HOC biodegradation by the indigenous sediment/soil microbiota.

Biochar can not only reduce HOC bioavailability in sediments/soils but also affect the fate of metabolites. Since HOC are biologically active molecules, they can potentially exert toxicity to non-target organisms within the sediment. Some HOC have been shown to have a range of short-term impacts on sediment/soil microbial communities, including reduction of biomass and altered community composition and function^7^. Since biochar reduces HOC bioavailability in sediments/soils, an application of biochar to sediments could reduce ecotoxicological impacts of HOC. Additionally, the effects of biochar on microbes have shown that biochar provides microbial refuges or nutrients to improve sediment microbial biomass and/or microbial activity due to its porous nature and abundance of the elements of C, N and P^8, 9^. Thus, the implication of biochar on HOC biodegradation in sediment involves two functions as it impacts HOC bioavailability and microbial activity. However, how the impacts of biochar occur on both HOC bioavailability and microbial activity in sediment is not clear.

Previous studies on the effects of biochar on the biodegradation of HOC focused mainly on HOC bioavailability^3–5^. For example, Rhodes *et al*.^5^ found that activated carbon (AC) depressed the mineralization of phenanthrene (PHE) in soil due to desorption inhibition. Yang *et al*.^4^ studied the effect of adding coal-derived AC to soil on PHE bioavailability to inoculated *Mycobacterium vanbaalenii* PYR-1. Compared to the unamended soils, the addition of AC at amounts of between 0.5% and 6% led to an increasing reduction in the amounts mineralized. However, there are only a few studies for using amendment in biochar matrix to impact HOC catabolic activity of the indigenous sediment/soil microflora. In those studies, the amendment showed positive effects of biochar on microbial degradation^10, 11^. For example, Bushnaf *et al*.^11^ found that a 2.0% biochar amendment could accelerate polycyclic aromatic hydrocarbon (PAHs) biodegradation rate, while Vasilyeva *et al*.^10^ reported that AC amendment accelerated biodegradation of 3,4-dichloroaniline (DCA) in sandy soils. AC promotes strong binding through accelerated microbial reduction of its nitro groups, catalytic chemical oxidation of the methyl group, and polymerization or binding of the products formed of 2,4,6-trinitrotoluene (TNT), due to a sharp reduction of the toxicity of these contaminated soils to microbes.

Previous research results concerning the impact of biochar on HOC biodegradation were contradictory. Overall, the impact of biochar on HOC biodegradation varied with different biochar percentages, HOC concentrations and microbial activity. Hence, in this study, the effects of rice straw biochar (RC) on nonylphenol (NP) biodegradation in sediment were investigated. In addition, aqueous available NP concentration, NP toxicity to microbes, microbial biomass/activity and microbial community structure were measured to explore impact of RC on NP availability and microbial ecology. To our knowledge, this is the first study to extensively investigate the role of biochar in NP biodegradation, and to understand the interactions among biochar, contaminants, and degrading microorganisms in sediments.

## Results and Discussion

### Biodegradation of NP in RC-sediment systems

**Figure 1:**
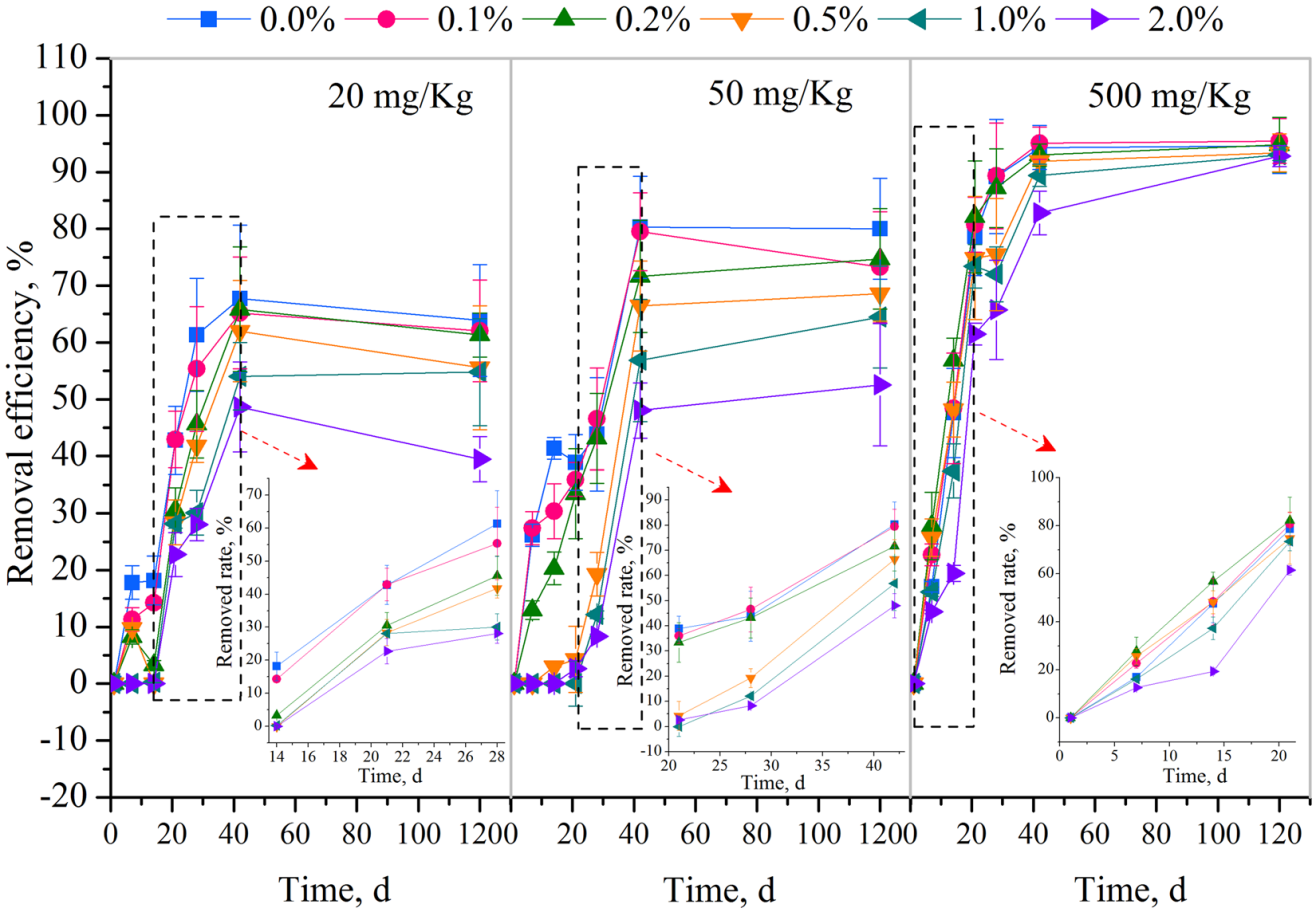
NP biodegradation curves in RC-sediment system with different RC percentages. NP rapid biodegradation period were marked with a dotted frame and showed in an enlarged figure for each NP concentration.

The biodegradation of NP with starting concentrations of 20, 50 and 500 mg/Kg after a 120 d incubation period at 25 °C in the sediments with 0.0%, 0.1%, 0.2%, 0.5%, 1.0%, and 2.0% RC are shown in Fig. 1. There was a rapid biodegradation period during the NP biodegradation process (marked with a dotted frame and showed in an enlarged figure for each NP concentration in Fig. 1). In the rapid biodegradation stage, when the NP concentrations were at 20 and 50 mg/Kg, the NP biodegradation ratio decreased with the increase of RC percentages (*P* < 0.01), while when the NP concentration was at 500 mg/Kg, NP biodegradation ratios in 0.1%, 0.2% and 0.5% RC amended sediment samples were higher than in the pure sediment samples (*P* < 0.01), in which there was no addition of biochar. When the RC percentages were > 0.5%, NP biodegradation ratios also decreased with the increase of RC percentages (*P* < 0.01). However, with biodegradation processing into the slow biodegradation stage, the NP biodegradation ratios decreased with the increase of RC percentages (0.01 < *P* < 0.05) at 500 mg/Kg, at which, the biodegradation ratios were similar with those at low concentrations (20 and 50 mg/Kg).

### Impact of RC on biodegradation of NP in sediments

**Figure 2:**
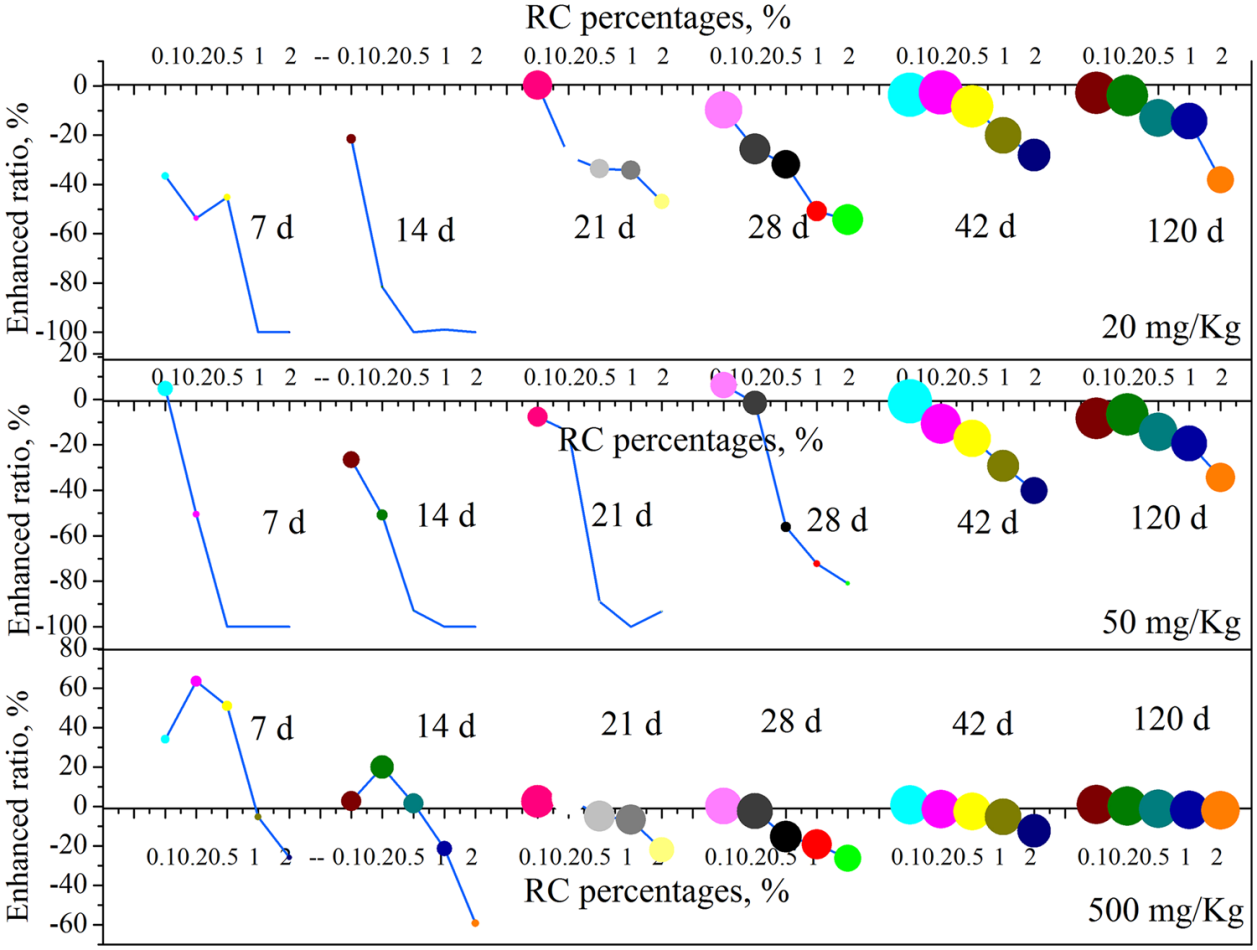
Impact of RC percentages on biodegradation of NP with different concentration in sediment. Enhancement ratio of RC on NP biodegradation was calculated by NP removal efficiency using pure sediment samples as blank control. 0.1, 0.2, 0.5, 1.0 and 2.0 in horizontal axis represent 0.1, 0.2, 0.5, 1.0 and 2.0% RC percentages in sediment, respectively. The size of color circles represents NP removal efficiency.

In order to examine the role of RC in NP biodegradation, an enhanced ratio of RC on NP biodegradation was calculated using pure sediment samples as blank controls (Fig. 2). Results showed that when NP concentrations were at 20 and 50 mg/Kg, the enhanced ratio of RC at different RC percentages on NP biodegradation were all negative, indicating that amendment of RC reduced NP biodegradation at low NP concentrations, and the extent of reduction increased with raised RC percentages. It has been reported that increased sorption of HOCs by carbonaceous materials, such as activated carbon, charcoal and biochar, might induce a decrease in microbial degradation due to a reduction in bioaccessibility and bioavailability^1, 2, 12, 13^. For example, a number of studies have demonstrated that even small amounts of a sediment/soil amendment, such as activated carbon, can reduce biodegradation due to its strong sorptive capacity and ability to increase the slowly and very slowly desorbing fractions leading to a reduced contaminant availability^3–5^.

When NP concentration was at 500 mg/Kg, the enhanced ratios of 0.1%, 0.2% and 0.5% RC amendment before 21 d on NP biodegradation were positive, suggesting that proper RC amendment promoted NP biodegradation at a high NP concentration. A similar result was observed by Vasilyeva *et al*.^10^ who found that 1.0% activated carbon could accelerate biodegradation of DCA and TNT with the high concentrations greater than 500 mg/Kg, by reducing the toxicity of readily available chemicals in soil and transferring them to a less toxic soil fraction. However, in this study, even at NP concentrations of 500 mg/Kg, a 1.0% or 2.0% RC amendment reduced NP biodegradation, like at low NP levels.

A pseudo first order kinetics model (Formula (1)) was applied to fit NP biodegradation curves. Fitted parameters are listed in Table 1. Both NP biodegradation fraction (*F*_de_) and average biodegradation rate constant (*k*_ave_) decreased, while half-life period (*t*_1/2_) (Formula (2)) increased with the increase of RC percentages at low NP concentrations (20 and 50 mg/Kg). This result indicated that RC suppressed NP biodegradation and slowed NP biodegradation, increased NP residue and extended NP residual time in sediment. At high NP concentration (500 mg/Kg), although *F*_de_ still trended to decrease with the increase of RC percentages, *k*_ave_ seemed increase from 0 to 0.2% RC, then decreased from 0.2 to 2% RC, and obtained its max *k*_ave_ at 0.2% RC. The half-life of NP first decreased then increased, and reached to lowest value at 0.2% RC, indicating that the proper proportion of RC accelerated NP biodegradation at high NP concentration.

**Table 1:** Fitted parameters of biodegradation curves of NP in RC-sediment system.

NP concentration	Parameters	0.0%	0.1%	0.2%	0.5%	1.0%	2.0%
20 mg/Kg	*F*_de_ (%)	70.10	67.90	68.67	62.95	61.22	42.09
	*k*_ave_ (%/ d)	0.047	0.044	0.032	0.031	0.026	0.027
	*t*_1/2_ (d)	14.88	15.83	22.00	22.15	26.76	25.77
	*R* ^2^	0.657	0.658	0.609	0.552	0.643	0.582
50 mg/Kg	*F*_de_ (%)	83.02	79.18	80.97	81.94	77.12	72.99
	*k*_ave_ (%/ d)	0.040	0.038	0.030	0.025	0.023	0.021
	*t*_1/2_ (d)	17.55	18.39	23.42	27.56	29.62	33.65
	*R* ^2^	0.891	0.742	0.930	0.634	0.583	0.624
500 mg/Kg	*F*_de_ (%)	101.57	101.80	99.00	97.17	98.06	98.68
	*k*_ave_ (%/ d)	0.055	0.058	0.066	0.056	0.047	0.035
	*t*_1/2_ (d)	12.56	11.99	10.53	12.45	14.87	19.92
	*R* ^2^	0.929	0.945	0.967	0.972	0.936	0.907

### Mechanism of RC affecting biodegradation of NP in sediments

#### Effects of RC on NP concentrations in water soluble fraction of RC-sediment system

**Figure 3:**
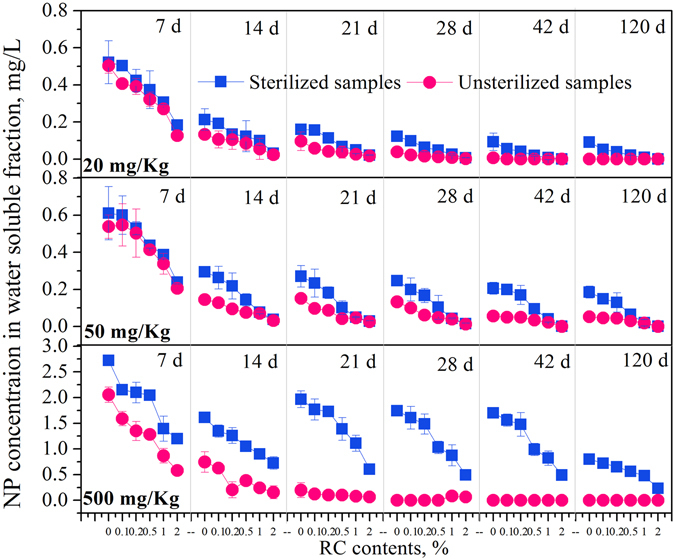
Changes of NP concentration in water soluble fraction with time in RC-sediment systems.

Previous studies indicated that only bioavailable contaminant can be utilized by organisms^14, 15^ and it had been suggested that the free state of HOCs might provide a direct measure of the microbial degradable contaminant fraction^16–18^. NP concentrations in the water soluble fraction in sediments with different RC percentages were measured to examine the effects of RC on NP bioavailability (Fig. 3). For the sterilized samples, NP concentrations in water soluble fraction decreased with increasing RC percentages in all abiotic samples, indicating that RC reduced NP bioavailability due to its high affinity to NP. The difference between sterilized and unsterilized samples, considered as the amount of NP biodegradation in the water soluble fraction, tended to decrease with increasing RC percentages in all samples, indicating RC depressed aquatic NP biodegradation by reducing NP bioavailability. In other studies, the application of carbonaceous amendments, such as AC, to sediment/soils also reduced both the rates and extent of HOC biodegradation due to the strong sorption^3, 5^. Although the fact that strong affinity of RC to NP reduced free state NP (Fig. 3), it is worth noting that proper RC amendment promoted NP biodegradation at high NP concentrations **(**Fig. 2).

#### Effect of RC on NP toxicity to microbes

**Figure 4:**
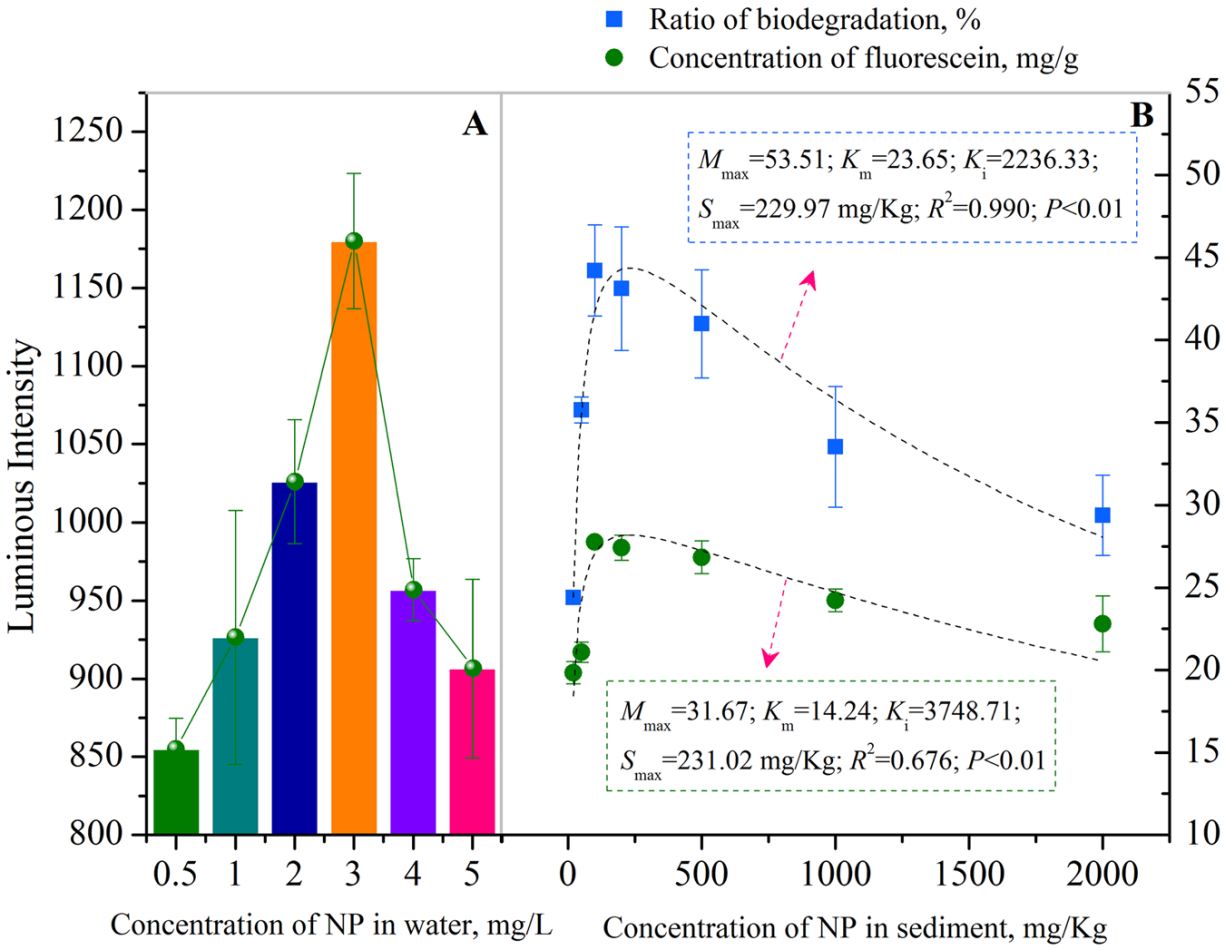
Toxicity of NP in aquatic phase and sediment phase respectively. (**A**) NP toxicity in aquatic phase to luminescent bacteria; (**B**) NP toxicity in sediment phase to FDA hydrolytic activity and NP biodegradation ratio.

Since biochar reduced HOC bioavailability in the sediments mentioned above, the application of biochar to sediments could also reduce the toxicity of NP to organisms. To test this hypothesis, the toxicity of NP in the aquatic phase and sediments were both estimated using the luminescent bacterial toxicity testing method (Fig. 4A) and FDA hydrolytic activity assay (Fig. 4B). The luminous intensity of the aquatic phase increased for NP < 3.0 mg/L, and decreased for NP > 3.0 mg/L, indicating NP stimulated luminescent bacteria while NP concentrations were lower than 3.0 mg/L and NP was toxic to luminescent bacteria while NP concentrations were higher than 3.0 mg/L in aquatic phase. And in the biodegradation system, initial water soluble NP concentrations were 0.50, 0.54 and 4.52 mg/L for pure sediments with the NP concentrations of 20, 50 and 500 mg/Kg, respectively, suggesting that when the NP concentrations in sediments were low (20 and 50 mg/Kg), initial water soluble NP concentrations were lower than 3.0 mg/L, so the RC amendment reduced stimulation of NP to bacterial activities. When the NP concentration in sediment was high (500 mg/Kg), NP initial water soluble concentration in the pure sediment was 4.52 mg/L. The NP concentration was 3.55 and 2.98 mg/L in 0.1% and 0.2% RC amendment, respectively, and the NP concentrations were all lower than 3.0 mg/L in ≥ 0.5% RC amendments. Hence, the 0.1% and 0.2% RC amendment reduced NP toxicity to bacteria and improved bacterial activities at 500 mg/Kg in sediment. However, ≥ 0.5% RC amendment reduced or even depressed the stimulation of NP to bacterial activities in sediment.

In order to further examine the toxicity of NP to microbes, the impact of different concentrations of NP in the sediment phase on FDA hydrolytic activity and NP biodegradation ratios were examined (Fig. 4B). Both FDA hydrolytic activity and NP biodegradation ratios initially increased then decreased with increasing NP concentrations in sediments, indicating that there could be a threshold under which the increase of NP concentrations stimulated enzyme activity and promoted NP biodegradation, while above which NP caused toxicity to sediment microbes and depressed NP biodegradation. In order to get this threshold, the Haldane model was applied to fit these two sets of data. The fitted data showed that *M*_max_ was 53.51% and 31.67 mg/g for NP biodegradation rate and concentration of fluorescein, respectively with a corresponding NP concentration of 229.97 and 231.02 mg/Kg, respectively, or approximately 230 mg/Kg for both. Consequently, 230 mg/Kg was identified as the NP toxic threshold value to sediment microbes. When the NP concentrations were 20 and 50 mg/Kg in the sediments, which were lower than 230 mg/Kg, no toxic effect was observed to the microbes, but when the NP concentration was 500 mg/Kg in the sediments, the NP was toxic to sediment microbes. A similar result was also observed that while azoxystrobin reduced dehydrogenase activity, black carbon (BC) addition greatly increased dehydrogenase activity^6^. However, even though 500 mg/Kg NP exhibited toxicity to microbes, both the NP biodegradation ratio and concentrations of fluorescein were higher than when the NP concentrations were at 20 and 50 mg/Kg.

#### Effect of RC on microbial quantity and activity in sediment

The qPCR method and FDA hydrolytic activity assay were applied to measure microbial quantities for all bacteria and enzymatic activity for biodegradation samples at 1, 21 and 120 d, respectively (Supplementary Figure S1). In the 21 d samples, when NP biodegradation rate was fastest (Supplementary Figure S2), both microbial quantities and enzymatic activity were higher than that in the 1 d and 120 d samples. This finding indicated that NP biodegradation can be influenced by microbial quantities and enzymatic activity. Microbial quantities and enzymatic activity in sediment samples with the NP concentration of 500 mg/Kg were higher than those in sediment samples with the NP concentrations of 20 and 50 mg/Kg. This suggests the NP concentration (500 mg/Kg) caused toxicity to microbes, but its stimulation was greater than that in low NP sediment (20 and 50 mg/Kg). This result is consistent with a study by Stenrad *et al*.^19^ and Jontofsohn *et al*.^20^. The former study found that NP promoted soil respiration, while the latter suggested that there were positive correlation between NP concentrations and bacterial cell numbers, and NP promoted increase of active bacteria. Similarly, it was found that the reduction of dehydrogenase activity by azoxystrobin was enhanced by BC addition^6^.

#### Effect of RC on microbial community in sediments

**Figure 5:**
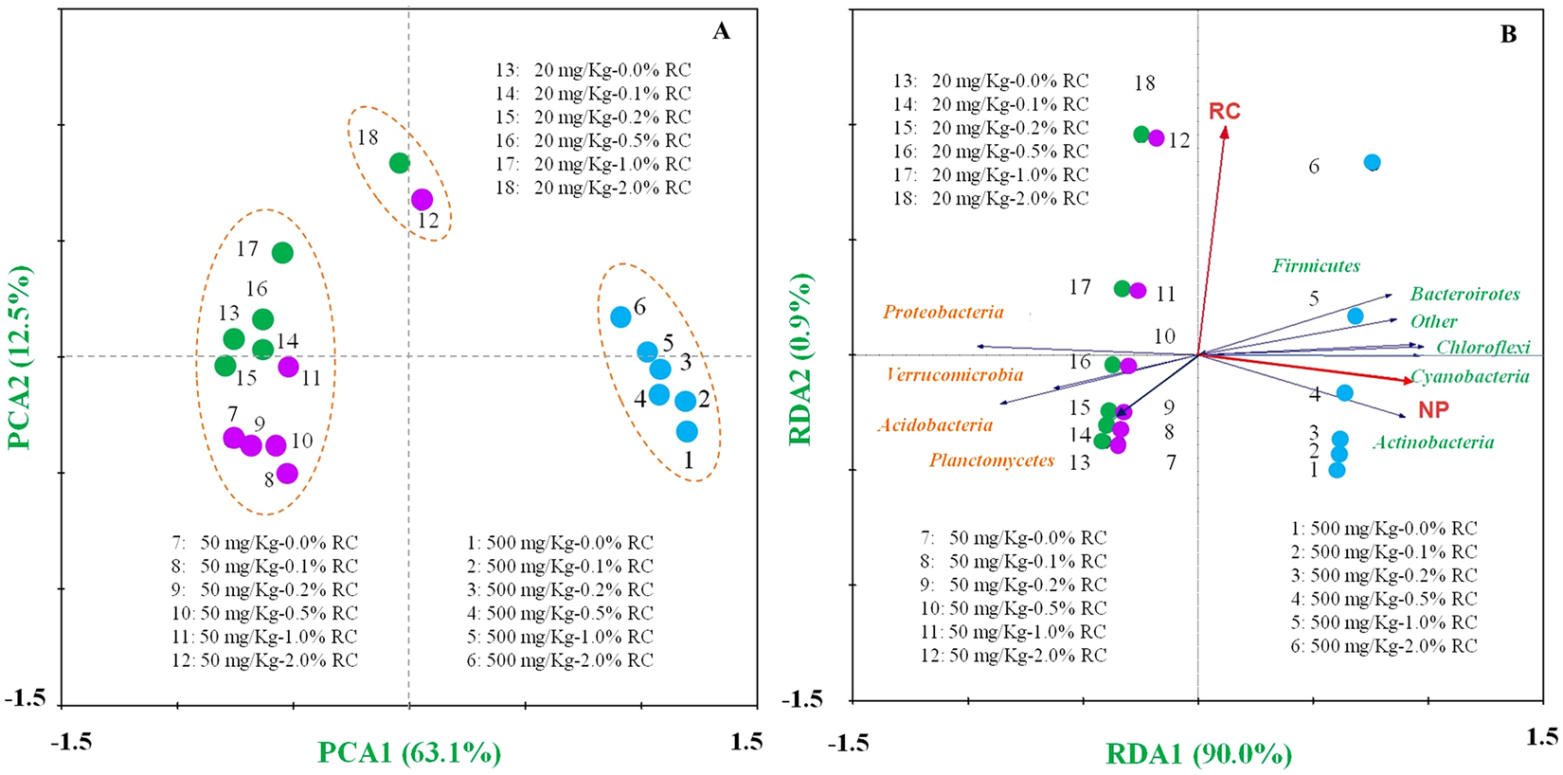
Cluster (**A**) and RDA (**B**) analysis at genus level.

Microbial community data in the pure sediment with different NP concentrations was illustrated in Supplementary Figure S3. The relationship of microbial community structures with NP concentrations and RC percentages was examined using PCA and RDA analysis at the genus level (Fig. 5). In the PCA analysis, 63.1% of the variance is explained by axis 1 and 12.5% by axis 2. Axis 1 separates microbial community structures into the 500 mg/kg NP group from the others (20 and 50 mg/kg NP), while axis 2 separates 2.0% RC amendment from < 2% RC amendment in the 20 to 50 mg/kg NP, indicating that NP was the main factor affecting microbial community structures. The first two axes of the RDA explained a high percentage of the variance (90.9%). The permutation tests confirmed that NP concentrations were significantly associated with the change in bacterial community structures (*P* < 0.01), which were in agreement with previous reports that low level NP amendment had no or little impact on aquatic bacterial community structures^19, 20^, but high level NP induced a significant shift in bacterial community structures^21^. For example, Stenrad *et al*.^19^ examined the impact of 10 mg/Kg NP on soil community structures and suggested that there was not a significant inhibition or enhancement on soil community structures by NP, and Jontofsohn *et al*.^20^ found 0.162–2.014 mg/Kg NP only slightly affected sediment microbial community structures. Wang *et al*.^21^ indicated that 150 mg/Kg significantly induced a shift of sediment microbial communities.

A number of studies previously suggested that biochar amendment could influence microbial community structure significantly^22–24^. For example, Tong *et al*.^24^, Anderson *et al*.^22^ and Khodadad *et al*.^23^ all found that biochar could improve relative abundance of dominant microbial species. However, in this study, there was no significant association of RC with the bacterial community composition (*P* > 0.05 for each comparison). RC could indirectly, rather than directly, affect sediment microbial community structures through the effect of RC on NP biodegradation, bioavailability and toxicity.

### Main functional microbial species in NP biodegradation system with RC amendment

**Figure 6:**
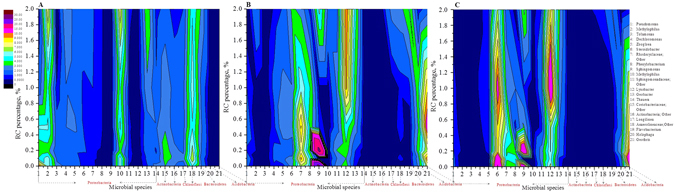
Microbe species (microbial relative abundance) responded with NP concentration and RC percentage. (**A**) NP concentration was 500 mg/Kg; (**B**) NP concentration was 50 mg/Kg; (**C**) NP concentration was 20 mg/Kg.

Taxonomic identity of each phylotype was determined using the Ribosomal Database Project (RDP) Classifier. A total of 264,922 trimmed sequences with the length of > 200 bp was obtained for 18 samples and 20476 operational taxonomic units (OTUs) with 97% similarity were identified. Microbial relative abundance ( > 1%) was compared at genus level among NP concentrations and RC percentages (Fig. 6). The concentrations of NP induced a significant shift in bacterial community during incubation. The relative abundance of some bacterial species increased with NP concentrations, such as some of Proteobacteria (*Pseudomonas*, *Methylophilus*, *Tolumonas*, *Dechloromonas* and *Zoogloea*), Chloroflexi (*Longilinea*) and Bacteroidetes (*Flavobacterium*). Furthermore, when the NP concentration was 500 mg/Kg, relative abundance of *Pseudomonas* was highest, while when NP at low level (20 and 50 mg/Kg), relative abundance of *Pseudomonas* was low. Previous studies reported that *Pseudomonas* isolated from NP contaminated sediments as NP degrading bacteria and NP was considered as energy and carbon sources for this bacteria^25, 26^. Wang *et al*.^21^ suggested that tolerance of Gammaproteobacteria, which the genus *Pseudomonas* belongs to, to NP was higher than 150 mg/Kg. In this study, NP concentration was up to 500 mg/Kg, indicating that the tolerance of *Pseudomonas* to NP could be as high as 500 mg/Kg. Thus, it is reasonable to infer that *Pseudomonas* was the predominant degrading bacteria at high NP levels. The relative abundance of some bacterial species decreased with increasing NP concentrations, such as some Proteobacteria (*Steroidobacer*, *Phenylobacterium*, *Sphingomonas*, *Methylibium* and *Lysobacter*), Acidobacteria (*Holophaga* and *Geothrix*). The relative abundance of *Sphingomonas* was up to 30% at low levels of NP, but decreased at high levels of NP. Previous studies reported that *Sphingomonas* could be isolated from NP contaminated sediments as NP degrading bacteria and those bacteria were considered to use NP as energy and carbon sources^27, 28^, suggesting that *Sphingomonas* might be the dominant degrading bacteria at low NP concentrations. So the genera of *Pseudomonas* and *Sphingomonas* were speculated as the predominant degrading bacteria at high and low NP concentrations, respectively.

In addition to *Pseudomonas* and *Sphingomonas*, bacteria *Flavobacterium* (Bacteroidetes) and *Lysobacter* (Gammaproteobacteria) are known for degradation of a variety of phenolic pollutants. *Flavobacterium* have been linked to degradation of pentachlorophenol and 3-methyl-4-nitrophenol^29^, while *Lysobacter* species are able to degrade trichlorophenol andpentachlorophenol^30^. Therefore, the presence of *Flavobacterium* and *Lysobacter* might have specific roles in NP biodegradation in sediments, which were in line with results of Wang *et al*.^21^.

Although this study did not demonstrate that the RC amendment impacted the sediment microbial community structure, the relative abundance of some bacterial species, such as Proteobacteria *Dechloromonas*, *Zoogloea*, *Lysobacter* and *Geobacter*, Chloroflexi *Longilinea* and Bacteroidetes *Flavobacterium* increased with RC percentages, respectively. While for some bacteria like Proteobacteria *Pseudomonas*, *Tolumonas* and *Steroidobacer*, their relative abundance decreased with RC percentage. Nonetheless, it was found that for some bacterial species like Proteobacteria *Dechloromonas* and *Zoogloea*, Chloroflexi *Longilinea* and Bacteroidetes *Flavobacterium*, their relative abundance increased with the increase of both NP concentrations and RC percentages, suggesting that both NP and RC affected those bacterial populations. Unlike the above mentioned bacterial populations, gamma proteobacteria *Lysobacter* trended to decrease with NP concentrations, but increase with RC percentage, which suggest that high NP concentrations may be toxic to them and/or inhibit their growth, while high RC amendments reduced NP toxicity.

### Role of RC in NP biodegradation in sediment

**Figure 7:**
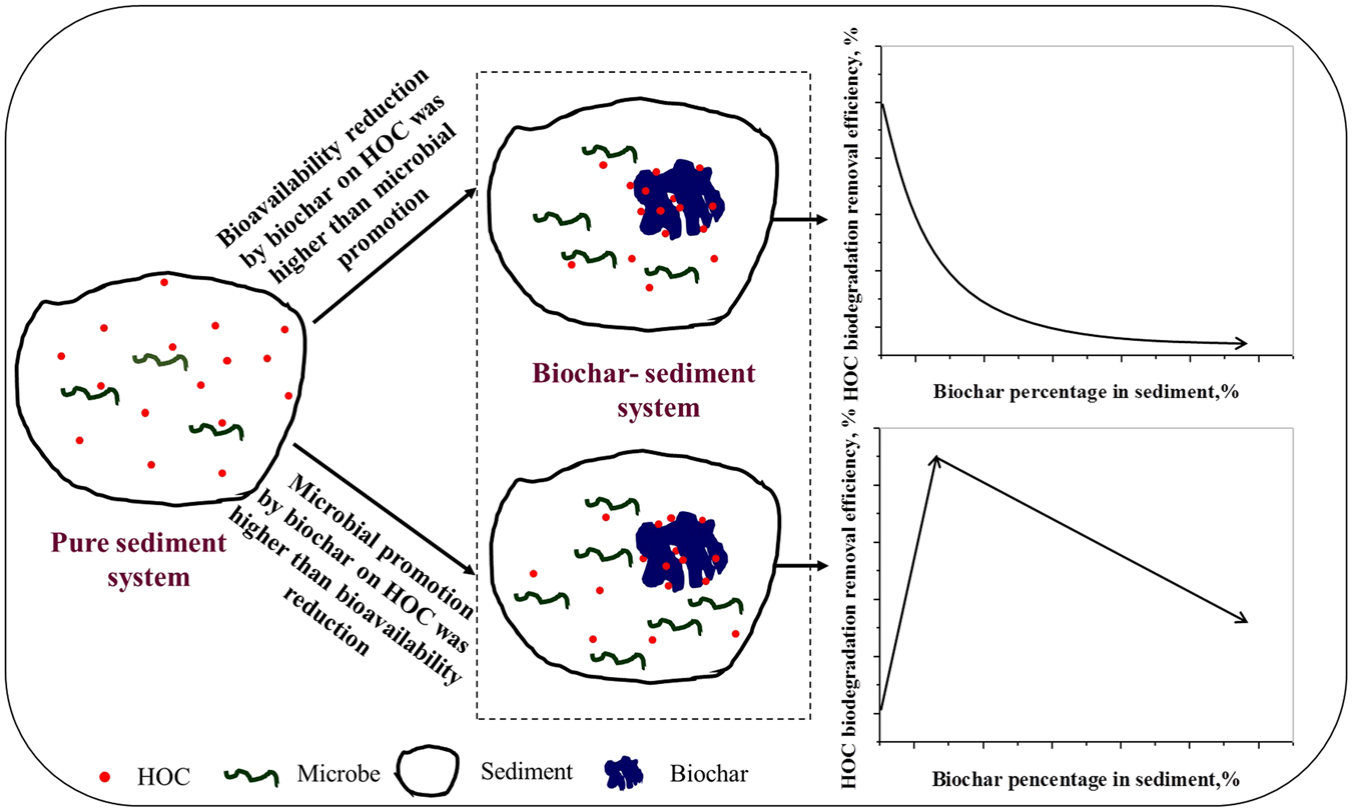
Scheme of role of biochar in NP biodegradation in sediment.

NP biodegradation in RC-sediment system is a comprehensive process, where NP, RC and microorganisms interacted and influenced each other. In this system, adsorption-desorption of NP and microorganisms to RC, microbial growth, and biodegradation of NP exist simultaneously. This study provides a new insight towards the effect of biochar on HOC biodegradation in sediments by studying combined impact of biochar on HOC bioavailability and microorganisms. The difference between impact intense of RC percentages on NP bioavailability and microorganisms determined whether RC reduced or promoted NP biodegradation (Fig. 7). Generally, at low level of NP, high RC amendment reduced NP bioavailability and stimulation of NP towards microorganism, resulting in reducing NP biodegradation. When NP concentration was higher than the max stimulating value to microorganism, an appropriate RC dosage amendment reduced NP toxicity to microorganism and promoted microorganism activity. The degree of increasing microbial activity of RC was more than extent of decreasing NP bioavailability and bioaccessibility, resulting in accelerating NP biodegradation. However, increasing microbial activity extent of large dosage RC to microorganism was less than extent of decreasing NP bioavailability and bioaccessibility, resulting in reduction of NP biodegradation. Overall, an appropriate biochar dosage amendment in HOC polluted sediment could form adsorption-biodegradation coupled remediation for organic pollution, which will intensify efficiency of organic pollution remediation.

## Conclusions

This study provides a new insight into effect of RC on NP biodegradation in sediments. Results showed that, the difference between impact of RC on NP bioavailability and microorganisms determined whether RC reduced or promoted NP biodegradation. Furthermore, RC affected microorganisms by changing NP toxicity and microbial quantity, activity, rather than by changing microbial community structure. This study indicated that there could be an optimal biochar percentage in biochar-sediment systems at different HOC concentrations when biochar was applied for organic pollution control, which strengthened HOC biodegradation process and accelerated biodegradation rate, forming adsorption-biodegradation coupled bioremediation.

## Methods

### Chemicals and materials

NP (Mixture of isomers) with a purity of > 99% was purchased from Aladdin (Shanghai, China) and prepared to generate a concentrated stock solution (100000 mg/L) with acetonitrile. Sediment was obtained from the Qiantang River, Hangzhou, Zhejiang province, China, using a clam sampler (Juchang Company, Qingdao, Shandong province). The sediment contained 55.01% of water, its pH was 7.14, its cation exchange capacity (CEC) was 10.94 cmol/kg, and its compositions of sand, silt and clay were 7.12%, 13.84% and 79.04%, respectively. The total organic carbon (TOC) and BC content of the sediment were 0.964% and 0.37%, respectively. The sediment sample used in this study did not contain a detectable quantity of NP.

The rice straw biochar was prepared from air-dried rice straw collected from the Hua-jia-chi Campus of Zhejiang University in China. The straws were burned on a stainless steel plate in an open field under uncontrolled conditions on a still and sunny afternoon^31^. The sample was treated in 2 M HCl and 1 M:1 M HCl-HF solutions to get purified biochar^31^. The treated rice straw biochar was oven-dried overnight at 105 °C, and recorded as RC. The characteristics of RC have been determined before, and are described in our previous work^31^. The sorption isotherms of NP by RC and RC-amended sediments were measured and all the sorption data were fitted well by Freundlich Model. Results indicated that RC posed high sorption capacity to NP with the Freundlich Model capacity factor *K*_*F*_ up to 43507.13 ± 1157.219 (mg/Kg)/(mg/L)^n^. Sorption details were described in our previous work^32^.

### Biodegradation experiment

The RC-amended sediments were prepared by mixing the sediments and specific quantities of RC to achieve amendment rates of 0%, 0.1%, 0.2% 0.5%, 1.0% and 2.0% (w/w). Sterilized Milli-Q water was added to keep same water content of all the samples. The RC-amended sediments were thoroughly mixed before spiking NP and conducting the biodegradation experiments. To assess NP loss by none biodegradation process, the same treated samples used as the controls were processed through sterilization at high temperature (121 °C) and high pressure (103 kPa,) for 20 min, and furthermore in order to inhibit bacterial growth, 0.2 mg/mL sodium azide (NaN_3_) was added throughout the whole incubation process.

#### Spiked and incubated experiment

Prior to biodegradation experiment, NP toxicity pre-experiment in sediment, involving in effect of NP toxicity on fluorescein diacetate (FDA) hydrolytic activity and NP biodegradation, was performed using NP concentrations at 20, 50, 100, 200, 500, 1000 and 2000 mg/Kg. Results showed that 230 mg/Kg in sediment was identified as the NP toxic threshold value to sediment microbes. Considering this threshold together with the NP concentrations of sediments (between several μg/Kg and hundreds mg/Kg dry weight) obtained from the previous investigations^33^, the selected two NP concentration ranges for biodegradation experiment were the concentrations lower than 230 mg/Kg (20 and 50 mg/Kg), which represented actual NP pollution conditions in the sediment, and the concentration higher than 230 mg/Kg (500 mg/Kg), which represented acute heavy NP sediment. In order to ensure the same acetonitrile volume in different treatments, NP stock solution (100000 mg/L) was diluted with acetonitrile into 4000 and 10000 mg/L, respectively. Then 50 g sterile or nonsterile RC-sediment mixtures were spiked with same volume (250 μL) of NP solutions with different concentrations (4000, 10000 and 100000 mg/L, respectively) to obtain the final concentrations of NP in sediment at 20, 50 and 500 mg/Kg, respectively. After mixing thoroughly, the mixtures in triplicate were incubated in the dark at room temperature for 1, 7, 14, 21, 28, 42 and 120 d, respectively.

#### Extraction and measurement of residual NP concentrations

NP concentrations were measured in aquatic and solid phase at different time intervals. Briefly, 1.0 g of the mixture was collected in a 50 mL glass centrifuge tube. 1 mL Milli Q-water was then added into each tube and vortexed for 1 min. The resultant slurries were centrifuged at 2500 rpm for 20 min. Anhydrous sodium sulfate was added into the glass centrifuge tube to remove residual water after the supernate was poured out. The sediments were extracted after sonication using 5 mL of methanol:methylbenzene mixture (6:1, v/v) in 50 mL glass centrifuge vials for each sediment sample^34, 35^. Sonication was performed for 40 min using a high-intensity ultrasonic processor (Boston Laboratory Equipment, Woburn, MA) three times. The three extracts from the same sample were combined and concentrated to 1 mL through nitrogen blowing.

#### NP analysis

NP concentrations were analyzed using high performance liquid chromatography (HPLC, Agilent 1100 series) with a diode array fluorescence detector (FLD) and a C18 reversed-phase column (ODS, 5 µm, 2.1 mm × 250 mm). Acetonitrile and water (70:30, v/v) were used as the mobile phase at the flow rate 1.0 mL/min, and the injection volume was 20 µL. The FLD wavelengths used for NP were the detection and excitation wavelengths 233 nm and 302 nm, respectively. The NP concentrations were quantified using an external standard. NP detection limit was about 1 µg/L.

#### Quality control

In the present study, quality control and safety measures were adopted to ensure reliability and validity of the results. First, prior to biodegradation experiment, the organic solvent extraction experiment was carried out to estimate extraction efficiency. Sediment and different RC amendment (0.5%, 1.0%, 2.0% and 5.0%) samples were extracted by sonication using 5 mL of methanol:methylbenzene mixture (6:1, v/v) across 4 repeated runs, and the NP recovery was 98.68 ± 3.965, 98.13 ± 2.678, 98.77 ± 1.991, 98.39 ± 2.780 and 97.62 ± 2.637% for sediment, 0.5%, 1.0%, 2.0% and 5.0% RC amendment, respectively, illustrating the practical effectiveness of the method. Second, the sterile sediment and RC controls were prepared to monitor NP recoveries. The abiotic losses of NP in the controls during the entire period were found less than 5.0%, which was considered to be negligible.

### Microbial toxicity experiment of NP

A luminescent bacterial toxicity testing method was used for aquatic NP toxicity determination^36^. For the test of NP toxicity in sediments, FDA hydrolytic activity assay and NP biodegradation were both used^36^. Briefly, the sediments were spiked with stocked NP to reach the final concentrations of 20, 50, 100, 200, 500, 1000 and 2000 mg/Kg, respectively. After that, the sediment samples treated in triplicate were incubated in the dark at room temperature for 14 d. Then, 2.0 g of the sediments was transferred into 125 mL Erlenmeyer flasks and 50 mL of sodium phosphate buffer and 0.50 ml of 4.9 mM FDA lipase substrate solution were added. The flasks were stoppered and swirled for a few seconds and then incubated for 3 h at 37–38 °C. To terminate FDA hydrolysis, two milliliters of acetone was added to the suspension and they were mixed by swirling flasks. About 30 mL of the sediment suspension was transfer to a 50 mL centrifuge tube and centrifuged at 5000 rpm for 5 min, and the supernatant was filtered through a Whatman No. 2 filter paper (ANPEL Scientific Instrument Co., Ltd., Shanghai, China). The filtrate was measured on a spectrophotometer set at a wavelength of 490 nm. To perform controls, the same procedures as described above were performed, but 0.50 mL acetone, instead of the fluorescein diacetate lipase substrate solution was added to the negative controls. Blanks were also included. The calculation of NP biodegradation ratios was through the extraction of the NP using 5 mL of methanol:methylbenzene mixture (6:1, v/v) in 50 mL glass centrifuge vials to assess NP toxicity on NP biodegradation.

### Microbial quantity, enzymatic activity and community structure

Microbial quantities were estimated using a quantitative PCR (qPCR) method for all bacteria according to the protocol described in previous studies^37^. Briefly, DNA was extracted from approximately 0.25 g of each sediment sample, with the MoBio Power Soil DNA Isolation Kit. DNA extraction was performed according to the manufacturer’s instructions. qPCR was performed using an iCycler iQ5 thermocycler (Bio-Rad, Berkeley, CA) in Personal Biotechnology Co., Ltd., (Shanghai, China). Each qPCR mixture (25 µL) was composed of 12.5 µL SsoFast EvaGreen Supermix (Bio-Rad, Berkeley, CA), 0.5 µL forward and reverse primers, and 1 µL template DNA. The qPCR thermal cycle program was performed as previously described^38^. Triplicate reactions of qPCR were performed for each sample and each dilution.

In order to get microbial community structure in sediments with different RC percentages and NP concentrations, 454 pyrosequencing was performed in Personal Biotechnology Co., Ltd., (Shanghai, China) with a set of primers (V3F: 5′-ACTCCTACGGGAGGCAGCAG-3′ and V4R: 5′-TACNVGGGTATCTAATCC-3′) targeting the hypervariable V3–V4 region (about 460 bp) of the 16 S rRNA gene. Equal amounts of purified amplicon products bearing individual 10 nucleotide barcodes from different samples were mixed for pyrosequencing on the Roche 454 FLX Titanium platform (Roche Diagnostics, Indianapolis, IN). The sequences were analyzed using the method described previously by Hao *et al*.^39^. Operational taxonomic unit (OTU), rarefaction curves and the diversity indices were determined using Mothur (www.mothur.org) and the taxonomy-based analysis was conducted using RDP-II Classifier of the Ribosomal Database Project (RDP) and the National Center for Biotechnology Information (NCBI) BLAST.

### Data analysis

SPSS for windows (Version 13.0) was used to statistically analyze NP biodegradation data. One-way ANOVA was applied to test difference of NP biodegradation data among different RC amendments with a 95% confidence level or α = 0.05.

#### NP biodegradation data fitted

Pseudo first order kinetics model was applied to fit NP biodegradation curves. The formula was as follows:

1 $${F}_{t}={F}_{de}+{F}_{de}\exp (-{k}_{ave}t)$$

Where *F*_t_ represents residual NP amount in RC-sediment system at time t, %; *F*_de_ represents biodegraded NP amount in RC-sediment system, %; *k*_ave_ represents NP average biodegradation rate constant, %/d.

The half-life period of NP in a RC-sediment system was calculated by the following formula:

2 $${t}_{1/2}=\,\mathrm{ln}\,2/{k}_{ave}$$

Where *t*_1/2_ represents half-life of NP in RC-sediment system, d.

#### Concentration Suppression Curve of NP

FDA hydrolytic activity and the ratios of NP biodegradation were plotted against time (t) and the Haldane model was fitted to the data, as follows:

3 $$V={V}_{{\rm{\max }}}/(1+{K}_{{\rm{m}}}/S+S/{K}_{{\rm{i}}})$$

4 $${S}_{{\rm{\max }}}=\sqrt{KmKi}$$

Where *V* represents the substrate reaction rate (instead of fluorescein concentration (mg/g) or NP biodegradation ratio (%) in this study); *V*_max_ represents the fastest reaction rate (the highest fluorescein concentration (mg/g) or NP biodegradation ratio (%) in this study); *K*_m_ represents the half rate constant; *K*_i_ represents the inhibition constant; *S* is the substrate concentration, mg/Kg; *S*_max_ is the corresponding NP concentration to the highest fluorescein concentration or NP biodegradation ratio, mg/Kg.

### Data Availability

All data generated or analysed during this study are included in this published article (and its Supplementary Information files).

## Electronic supplementary material

Supplementary information
