# Supplementary material for: Role of biochar in biodegradation of nonylphenol in sediment: Increasing microbial activity versus decreasing bioavailability
Source: Sci Rep. 2017 Jul 5;7:4726. doi: 10.1038/s41598-017-04787-2 (PMC5498626; doi:10.1038/s41598-017-04787-2)
Supplement: Supplementary file 1 — Supplementary information [file 41598_2017_4787_MOESM1_ESM.pdf]

Role of biochar in biodegradation of nonylphenol in sediment: Increasing microbial activity versus decreasing bioavailability

Guanghuan Cheng [a, b], Mingyang Sun [a], Jingrang Lu [c], Xinlei Ge [a], Huihui Zhang [b], Xinhua Xu [b], Liping Lou [b]*, Qi Lin [b]

[a] Jiangsu Key Laboratory of Atmospheric Environment Monitoring and Pollution Control (AEMPC), School of Environmental Science and Engineering, Nanjing University of Information Science &Technology, Nanjing 210044, People's Republic of China;

[b] Department of Environmental Engineering, Zhejiang University, Hangzhou 310029, People's Republic of China;

[c] Office of Research and Development, U.S. Environmental Protection Agency, Cincinnati, OH 45220, USA.

* Corresponding author, Tel.: +86-571-88982502. Fax: +86-571-88982502.
E-mail address: loulp@zju.edu.cn

Supplementary Information

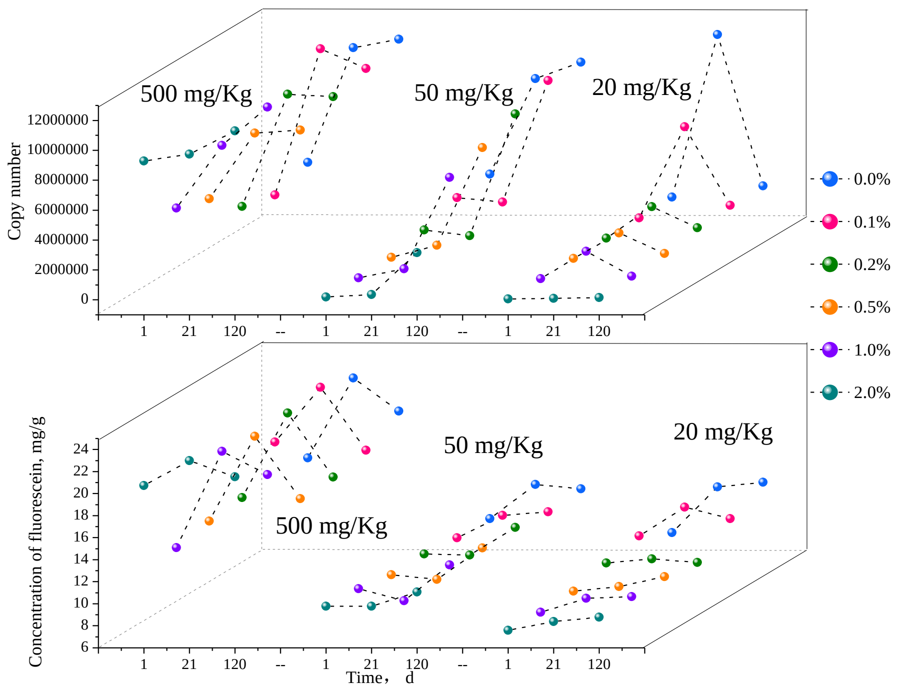

Supplementary Figure S1 The microbial quantity and FDA hydrolytic enzyme of in RC-sediment system at 1, 21 and 120 d; 20, 50 and 500 mg/Kg were NP concentrations in RC-sediment system, respectively. 0.0% -2.0% represent RC percentages in sediment, respectively

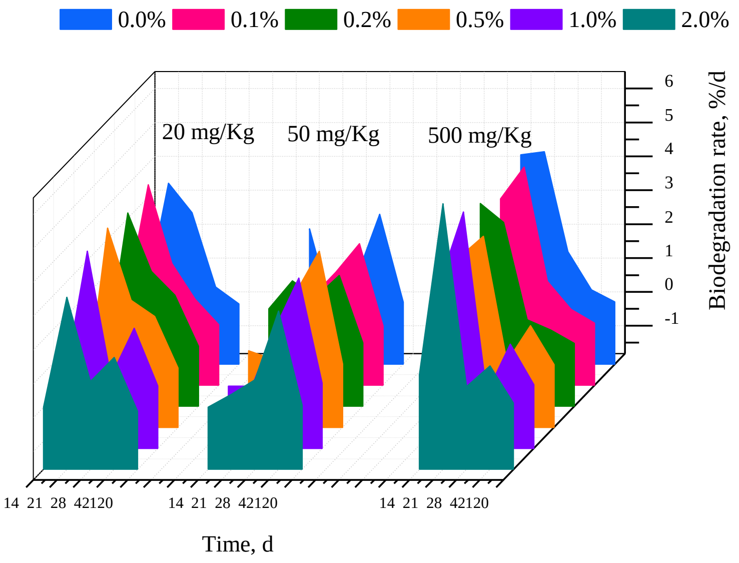

Supplementary Figure S2 Biodegradation rate of NP in RC-sediment system; 20, 50 and 500 mg/Kg were NP concentrations in RC-sediment system, respectively. 0.0% -2.0% represent RC percentages in sediment, respectively

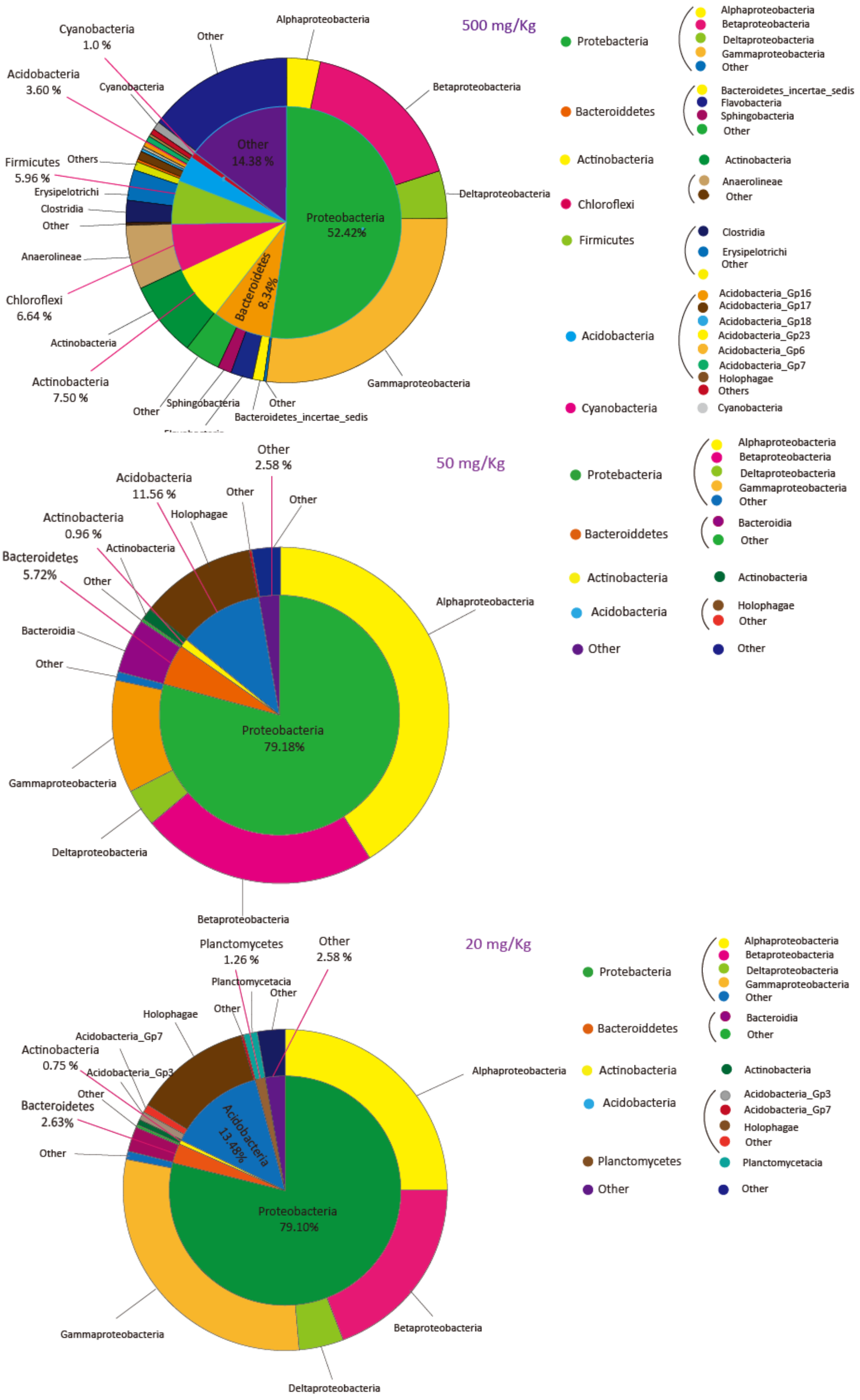

Supplementary Figure S3 Microbial diversity (Phylum) in pure sediment system without RC amendment with different NP concentrations (20, 50 and 500 mg/Kg)
